# Influence of magnetic field on a novel scintillation dosimeter in a 1.5 T MR‐linac

**DOI:** 10.1002/acm2.14180

**Published:** 2023-11-27

**Authors:** Stijn Oolbekkink, Bram van Asselen, Simon J. Woodings, Jochem W. H. Wolthaus, J. H. Wilfred de Vries, Adriaan A. van Appeldoorn, Marcos Feijoo, Madelon van den Dobbelsteen, Bas W. Raaymakers

**Affiliations:** ^1^ Department of Radiotherapy University Medical Center Utrecht Utrecht The Netherlands; ^2^ Blue Physics Lutz Florida USA

**Keywords:** MRI‐linac, radiotherapy, scintillation dosimetry, time‐resolved dosimetry

## Abstract

For commissioning and quality assurance for adaptive workflows on the MR‐linac, a dosimeter which can measure time‐resolved dose during MR image acquisition is desired. The Blue Physics model 10 scintillation dosimeter is potentially an ideal detector for such measurements. However, some detectors can be influenced by the magnetic field of the MR‐linac. To assess the calibration methods and magnetic field dependency of the Blue Physics scintillator in the 1.5 T MR‐linac. Several calibration methods were assessed for robustness. Detector characteristics and the influence of the calibration methods were assessed based on dose reproducibility, dose linearity, dose rate dependency, relative output factor (ROF), percentage depth dose profile, axial rotation and the radial detector orientation with respect to the magnetic field. The potential application of time‐resolved dynamic dose measurements during MRI acquisition was assessed. A variation of calibration factors was observed for different calibration methods. Dose reproducibility, dose linearity and dose rate stability were all found to be within tolerance and were not significantly affected by different calibration methods. Measurements with the detector showed good correspondence with reference chambers. The ROF and radial orientation dependence measurements were influenced by the calibration method used. Axial detector dependence was assessed and relative readout differences of up to 2.5% were observed. A maximum readout difference of 10.8% was obtained when rotating the detector with respect to the magnetic field. Importantly, measurements with and without MR image acquisition were consistent for both static and dynamic situations. The Blue Physics scintillation detector is suitable for relative dosimetry in the 1.5 T MR‐linac when measurements are within or close to calibration conditions.

## 1 INTRODUCTION

The MR‐linac is a combination of an MRI scanner and a linear accelerator, that can give high quality soft tissue contrast during treatment.^[^ ^1^, ^2^, ^3^ ^]^ With such a system, adaptive radiotherapy (ART) can be performed in which MRI is used to identify and correct for anatomical changes before and during the treatment.^[^ ^4^ ^]^ This anatomical information can be used for online plan adaptation,^[^ ^5^ ^]^ drift corrections,^[^ ^6^ ^]^ gating and tracking.^[^ ^7^, ^8^, ^9^ ^]^

Dosimetric quality assurance (QA) of ART plans is challenging as it requires a dosimeter that can be used during an adaptive workflow in which the treatment plan ultimately could change in real‐time. For example, when a target is irradiated using tracking.^[^ ^9^ ^]^ In addition, continuous MRI acquisition is required to obtain the anatomy real‐time. Therefore, an ideal detector for QA of real‐time ART in a magnetic field should fulfill the following properties: a dose response that is not affected by a magnetic field (while moving); the detector has a high sampling rate and spatial resolution; the ability to measure dose while MRI images are being acquired and the ability to accurately measure small fields including stereotactic treatments.^[^ ^10^, ^11^ ^]^

Plastic scintillation dosimeters (PSDs) potentially fulfill all of the mentioned properties.^[^ ^12^, ^13^, ^14^, ^15^ ^]^ PSDs are constructed out of non‐ferromagnetic materials, can be used safely inside the MRI and can be used to measure dose without mutual disturbance during MRI acquisitions. Scintillation detectors convert delivered dose to a linearly correlated light signal which can be read out. Combined with a high sampling rate and small detection volume, PSDs show great potential for time‐resolved dosimetry during MRI acquisitions without dose deviations and can therefore be used in QA of for example ART.^[^ ^16^, ^17^, ^18^ ^]^

In plastic optical fibers Cherenkov light is generated by decelerating, fast traveling charged particles inside the optical fiber. The measured signal of the PSD is therefore a combination of the scintillation signal and Cherenkov light. To eliminate the contribution of Cherenkov radiation inside the optical fibers, several techniques have been investigated such as chromatic filtering, spectral filtering and background subtraction.^[^ ^15^, ^19^, ^20^, ^21^ ^]^

Cherenkov radiation is mainly generated by high energy electrons traveling faster than the speed of light in that medium, and is emitted under a characteristic angle relative to the direction of the electrons. Without a magnetic field the electron fluence would be more or less rotationally symmetric around the beam axis in the center of the beam. In the presence of a magnetic field orthogonal to the photon beam direction, the electron trajectories are altered by the Lorentz force resulting in an asymmetrical electron fluence.^[^ ^22^, ^23^ ^]^ This implies that the resulting Cherenkov distribution is also changed. Therefore, the response of the detector for different orientations relative to the photon beam and magnetic field may be affected similar to the effects seen in an ionization chamber.^[^ ^24^ ^]^

The company “Blue Physics” developed PSD employing a Cherenkov subtraction‐based method for potential use in real‐time adaptive workflows on the MR‐linac. This PSD was characterized by Ferrer et al.^[^ ^25^ ^]^ In this characterization, the PSD was investigated for use in the 1.5 T MR‐linac for a single orientation with respect to the magnetic field, for both the calibration and the measurements. However, to use this detector in vivo or while in‐motion, the angular and radial dependence needs to be assessed.

The aim of this work is to evaluate the dependence of this PSD on different calibration methods within a magnetic field including the dependencies between angle of detector, radiation beam and magnetic field. The capability of the PSD to perform dynamic, time‐resolved measurements, including during MR image acquisition, will be demonstrated.

## 2 MATERIALS AND METHODS

### 2.1 Setup

Measurements were performed on a Unity 1.5 T MR‐linac (Elekta AB, Stockholm, Sweden). The MR‐linac has a 7 MV flattening filter free photon beam with an average dose rate of 420 MU per minute at a fixed source axis distance (SAD) of 143.5 cm. Reference measurements were performed on a Versa HD linac (Elekta AB, Stockholm, Sweden) with a flattened 6 MV photon beam and a dose rate of 508 MU per minute at an SAD of 100 cm.

#### 2.1.1 Blue Physics scintillator

The Blue Physics (Lutz, Florida, USA) scintillator, model 10, uses a BCF‐10 scintillation fiber manufactured by Saint Gobain (Hiram, Ohio, USA) which is 1 mm long, 1 mm in diameter and has a volume of 0.785 mm^3^. The core of fibers is manufactured out of polymethyl‐methacrylate with a fluoridated polymer cladding. The fibers are 0.25 mm in diameter and the complete fiber package is approximately 20 m long from detector tip to readout device. This PSD uses the subtraction method to remove the background signal from the scintillation signal. The system is capable of sampling at 1.4 kHz (700 µs).

#### 2.1.2 Subtraction method

**FIGURE 1:**
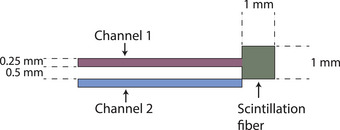
Schematic overview of the Blue Physics PSD. Channel 1 is connected to a scintillation fiber, channel 2 is identical to channel 1 without the scintillation fiber present. PSD, plastic scintillation dosimeter.

In a subtraction‐based Cherenkov removal technique, each detector uses two fibers connected to a dual‐channel transducer which converts light transported inside the optical fibers into charge (Figure 1).

The fibers are the same, but only one is coupled to the scintillation fiber, therefore the readings are as described in Equation (1):

(1) $$R_{1}=R_{1,S}+R_{1,C}\;and\;R_{2}=R_{2,C}$$

where the reading of channel 1 (*R*_1_) combines the scintillation signal ($R_{1,S}$) and the Cherenkov ($R_{1,C}$) induced signal. The reading of channel 2 (*R*_2_) only consists of Cherenkov light ($R_{2,C}$). In this subtraction technique, two identical fibers are used and therefore the assumption is that the ratio between the Cherenkov light is constant. The adjacent channel ratio (ACR) calibration factor ($k_{acr}$) reflects any differences in detection efficiency (i.e., optical coupling) in both fibers. The scintillation signal $R_{1,S}$ can therefore be expressed as:

(2) $$R_{1,S}=R_{1}-k_{acr}R_{2,C}\;with\;k_{acr}=R_{1,C}/R_{2,C}$$

Equation (2) can be used to calibrate the scintillation system by obtaining $k_{acr}$ with a set of measurements, in which the Cherenkov contribution in the fibers is changed while the scintillation signal remains the same, that is, the delivered dose to the scintillation fiber remains equal. Generally this is achieved by a set of measurements in which the amount of fiber exposed to the delivered field is changed.^[^ ^12^, ^13^, ^26^ ^]^

(3) $$D=k_{ctd}R_{1,S}$$

To obtain the dose, the reading of the scintillating fiber ($R_{1,S}$) is converted using a charge to dose conversion factor ($k_{ctd}$) (Equation 3). The $k_{ctd}$ is calibrated using a known dose measured with a reference chamber in BEAMSCAN MR water phantom (PTW, Freiburg, Germany, SN.171869) at isocenter with 10 cm water buildup using a 10 × 10 cm^2^ field (100 MU) at an SSD of 133.5 cm^2^.

During the study, several detectors are used for comparison. All of these detectors have been characterized and validated for use in the presence of a magnetic field. The detectors used are the Semiflex 0.125cc ionization chamber (PTW, Freiburg, Germany, SN. 7898, T31010),^[^ ^27^ ^]^ a microDiamond diode detector^[^ ^28^ ^]^ (PTW, Freiburg, Germany, SN.123759, T60019) and Farmer ionization chamber (PTW, Freiburg, Germany, SN.8377, T30013).^[^ ^24^ ^]^

### 2.2 Calibration methods

In all of the following calibration methods the RW3 slab phantom (PTW, Freiburg, Germany) is used unless stated otherwise.^[^ ^27^ ^]^ Measurements on the MR‐linac are performed with either 5 or 10 cm build‐up material with the detector surrounded by water to prevent air cavities.^[^ ^29^ ^]^ The reference measurements on the conventional linac are performed with 5 cm build‐up material. In all calibration methods the detector is positioned at isocenter and irradiated from gantry angle 0°. In this paper the IEC61217 convention is used.^[^ ^30^ ^]^

**FIGURE 2:**
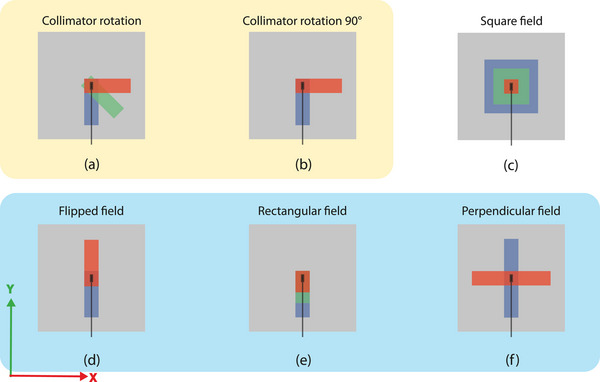
A schematic overview of all the $k_{acr}$ calibration methods. The methods are divided into three groups: calibration methods performed only on the conventional linac (a) and (b). Calibration methods performed only on the MR‐linac (c). Calibration methods performed both on the conventional and MR‐linac (d), (e), and (f). Field apertures are schematically illustrated in red, green and blue.

A common calibration technique for the PSDs at conventional linacs uses a fixed field aperture and number of MUs for two or more different collimator angles (Figure 2a).^[^ ^13^, ^14^, ^15^ ^]^ This method will be referred to as the ‘collimator rotation’ method. The $k_{acr}$ obtained using only two collimator angles (Figure 2b) is called the ‘collimator rotation 90°’ method. A field aperture of 4 × 13 cm^2^ (x1 = x2 = 2 cm, y1 = 11 cm and y2 = 2 cm) is used.

The above mentioned calibration methods are not possible on the MR‐linac since the collimator of the MR‐linac cannot be rotated. Therefore, a new calibration method is required using the same principle for determining $k_{acr}$ by changing only the Cherenkov contribution (Figure 2c–f). The proposed calibration methods, as described below, have a setup in which the detector is mounted in the +y direction, antiparallel to the magnetic field.

The ‘square field’ method (Figure 2c) uses multiple square field apertures. The detector is mounted in the BEAMSCAN MR water phantom and positioned at isocenter at a depth of 10 cm. The field aperture is varied from 4 × 4 cm^2^ (x1 = x2 = y1 = y2 = 2 cm) up to 15 × 15 cm^2^ (x1 = x2 = y1 = y2 = 7.5 cm) and delivered from gantry angle 0°. An equal delivered dose is obtained by correcting the MU with the relative output factor (ROF), which is verified with a Semiflex 0.125cc detector. A similar calibration method is described by Ferrer et al.^[^ ^25^ ^]^

The ‘flipped field’ method (Figure 2d) uses two asymmetric field apertures in which the y1 and y2 of the MLC are flipped for the two measurements. Two 4 × 13 cm^2^ field apertures are used (x1 = x2 = 2 cm with y1 = 11 cm, y2 = 2 cm, and with y1 = 2 cm, y2 = 11 cm).

The ‘rectangular field’ method (Figure 2e) uses a fixed field aperture of 4 cm (x1 = x2 = 2 cm) and various lengths 6, 8, 10, and 13 cm (y2 = 2 cm) along the ‐y‐axis (by changing y1) to include more fiber. Dose corrections are based on reference measurements using a Semiflex 0.125cc chamber to compensate for the change in output.

Finally, the ‘perpendicular field’ method is investigated (Figure 2f). In this method two perpendicular field apertures are used in which the sides of the fields are transposed (22 × 4 cm^2^ (x1 = x2 = 11 cm, y1 = y2 = 2 cm) and 4 × 22 cm^2^ (x1 = x2 = 2 cm, y1 = y2 = 11 cm) using the same MU and dose, which is validated using a Semiflex 0.125cc ionization chamber.

In all MR‐linac experiments, the combined average of the $k_{acr}$ values of the various calibration methods will be used to determine the relevant quantities such as the dose. The sensitivity in the various dosimetric quantities due to variation in $k_{acr}$ is reported as $u_{acr}$. The $u_{acr}$ is the deviation in readout with the minimum and maximum values of $k_{acr}$.

### 2.3 Basic characterization

The performance of the PSD is characterized by measuring the dose reproducibility, dose linearity, dose rate dependence, percentage depth dose (PDD) curve, ROFs and axial dependency. These measurements are performed using the PTW BEAMSCAN MR water phantom. The scan range of the water phantom is 568 × 145 × 355 mm^3^ (width × height × length).^[^ ^31^ ^]^ For the water phantom setup a plate with ball‐bearings is positioned at the base of the water phantom, and its MV projection is imaged using the electronic portal imaging device. Using the PTW QA alignment software, any rotation of the water phantom itself is corrected. After this, ball bearings are mounted on the detector holders and used to correct for translations and rotations of the measurement axis. Tilts of the water phantom and its axis are corrected using the water sensor. Unless specified otherwise, all characterization measurements are performed in this setup using a field aperture of 10 × 10 cm^2^ delivered from gantry angle 0°, 100 MU per beam with the detector positioned at isocenter at 10 cm depth, antiparallel to the magnetic field and perpendicular to the delivered beam. All readings are normalized to the detector reading measured prior to the start of that experiment unless otherwise specified. A RW3 slab phantom is used to measure the radial response of the detector with respect to the magnetic field.

For the short term and medium term dose reproducibility, the maximum deviation with respect to the mean reading is determined. The short term dose reproducibility is assessed with 10 consecutive measurements. Medium term dose reproducibility is assessed over the course of two days with each measurement performed twice and averaged.

Dose linearity measurements are performed for a range of 1 MU up to 2000 MU's for both the scintillation detector and Farmer ionization chamber. All measurements up to and including 200 MU are performed twice and averaged. The 500, 1000, and 2000 MU measurements are performed once. Readouts are rescaled to 100 MU and compared to the ionization chamber results.

The dose rate dependence of the detector is evaluated by changing the gun duty cycle of the linac, resulting in dose rates of 50, 126, 252, 405, 420, 455, and 505 MU/min. All measurements are performed twice, averaged and compared to the measured readout of the nominal dose rate 420 MU/min.

PDD curves are measured along the ‐z‐axis ranging from −3 mm to +130 mm. The PDDs are measured for the PSD and a microDiamond detector (positioned antiparallel to the beam) used as a reference detector. PDD measurements for the scintillator are performed twice for each depth (50 MU per beam) and normalized to Dmax. Readings at the same depth are averaged and compared to the microDiamond results.

ROFs are measured for field apertures ranging from 0.5 × 0.5 cm^2^ up to 57.4 × 22.0 cm^2^ with both the scintillation detector and microDiamond. Scintillator measurements are performed twice and averaged. Measurements with the microDiamond are performed once. Additionally, for small fields (⩽ 2.0 × 2.0 cm^2^) correction factors according to the IAEA TRS‐483 for conventional 6 MV beam with no magnetic field are used.^[^ ^28^, ^32^, ^33^ ^]^

### 2.4 Response of detector with respect to the magnetic field

#### 2.4.1 Axial rotation of detector

**FIGURE 3:**
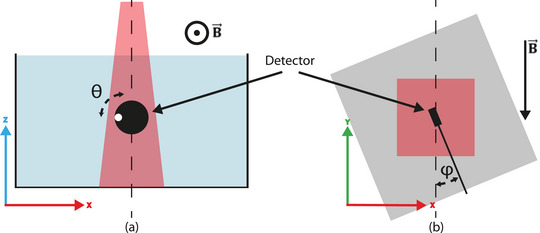
Schematic overview of the measurement setup (not to scale) for: (a) the axial response of the detector (angle Θ) where the white dot represents a reference marker on the detector, and (b) radial response (angle φ) of the detector with respect to the magnetic field.

The effect of axial rotation around the axis of the fiber is assessed (Figure 3a). The detector is positioned at isocenter at a depth of 10.0 cm and is rotated around its axis with increments of 90°, each angle θ is measured three times and averaged. A marking on the detector was used as reference, as schematically illustrated in Figure 3a. A 5 × 5 cm^2^ field aperture is used. All measurements are normalized to the 0° measurement.

#### 2.4.2 Rotation relative to magnetic field

The effect of the scintillation detector orientation with respect to the magnetic field on the response is also assessed. The scintillator is positioned at isocenter with 5 cm of build‐up in a RW3 slab phantom. The phantom including the detector is rotated with angle φ around the z‐axis (Figure 3b) with increments of 22.5° spanning 360°. Detector readings are normalized to the measurement at angle 0°.

### 2.5 The effect of MRI RF pulses and dynamic measurements

The effect of MRI acquisitions (RF pulses) during dose delivery on the detector response is assessed using the QUASAR MRI^4*D*^ motion phantom (Modus Medical Devices inc., London, Ontario, Canada) (SN. 1009, 1498, 4489). This phantom allows for movement during irradiation and MR imaging.

To test the effect of MRI RF pulse dependency a measurement is performed with the insert, in which the detector is mounted, at the central position of the phantom. Four beams with apertures of 10 x 10 cm^2^, 50 MU each, for gantry angles 45°, 135°, 225°, and 315° are used. Measurements are performed with and without MR image acquisition. During this static measurement, the insert with detector is not moved.

Dynamic measurements are performed with and without acquisition of MRI images. For these measurements the same plan is used as for the static measurements. A sinusoidal motion pattern along the y‐axis of 0.2 Hz (12 bpm) and an amplitude of 20 mm is used (see Figure 7a).

**FIGURE 4:**
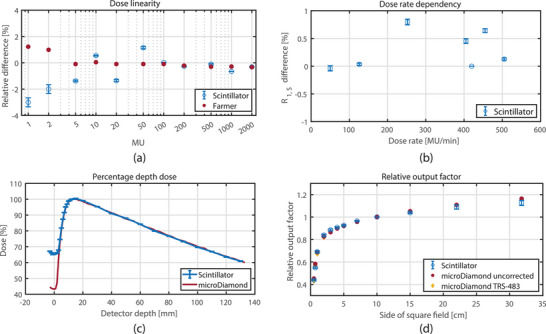
Results of the characterization measurements of the detector. The error bars represent only the variation due to potential calibration differences ($u_{acr}$).

**FIGURE 5:**
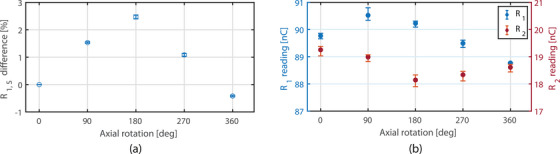
(a) Axial rotation dependency of the detector. The error bars represent only the variation due to potential calibration differences ($u_{acr}$). (b) Raw readings showing a difference between channel 1 (*R*_1_, left) and 2 (*R*_2_, right). Error bars represent the maximum and minimum measured values.

**FIGURE 6:**
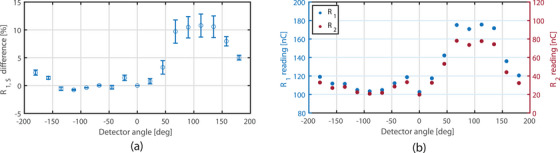
(a) $R_{1,S}$ as a function of radial angle between detector and magnetic field. The error bars represent only the variation due to potential calibration differences ($u_{acr}$). (b) The reading of channel 1 (*R*_1_, left) and 2 (*R*_2_, right).

**FIGURE 7:**
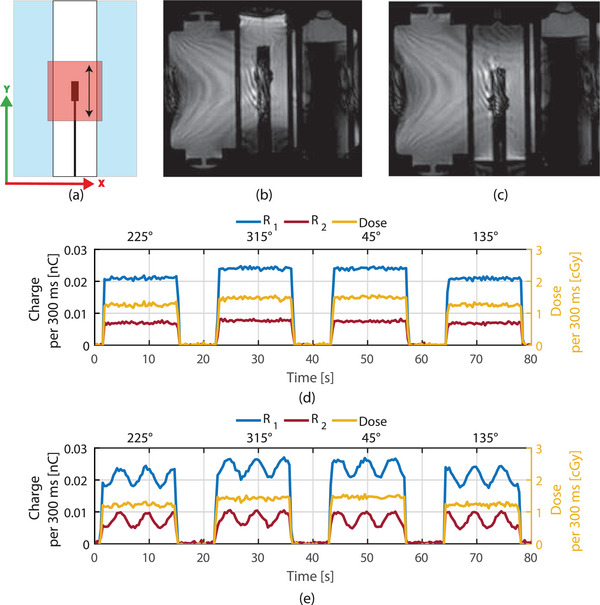
Static and dynamic measurements performed with the detector during cine MRI acquisitions. (a) the schematic movement of the insert is shown. (b) and (c) show the MR image acquired of the insert at maximum amplitudes. (d) Static reference and (e) dynamic measurements of both channels and dose, down sampled to 300 ms.

## 3 RESULTS

### 3.1 Calibration methods

The averaged $k_{acr}$ calibration values obtained for the different calibration methods (from Figure 2) are shown in Table 1. The variation between the different methods was larger for the MR‐linac compared to those of the conventional linac. Furthermore, on average the $k_{acr}$ is lower for the MR‐linac compared to the conventional linac. The two‐field methods on the MR‐linac show a higher $k_{acr}$ than the multi‐field methods. Additionally, the $k_{acr}$ obtained using two‐field methods on the MR‐linac showed a closer resemblance to the $k_{acr}$ obtained on the conventional linac. The average of all the MR‐linac $k_{acr}$ values was 1.11 and is used in the remainder of the paper. The lowest $k_{acr}$ (1.08) and the highest $k_{acr}$ (1.14) were used to demonstrate the calibration‐dependent variation in dosimetric quantity ($u_{acr}$) (see Figures 4, 5 and 6).

**TABLE 1:** The mean $k_{acr}$, for each of the different calibration method (from Figure 2).

	MR‐linac			Conventional linac
Method	$k_{acr}$	*n*	Max deviation	$k_{acr}$
Collimator rotation	‐	‐	‐	1.14
2 Field collimator rotation	‐	‐	‐	1.14
Square field	1.09	3	0.01	‐
Flipped field	1.13	2	0.01	1.15
Rectangular field	1.10	2	0.01	1.13
Perpendicular field	1.13	2	0.01	1.14

*Note*: The table also shows the number of repetitions of the measurements (*n*) and the maximum difference of the $k_{acr}$ with respect to the mean for that method. For the conventional linac all measurements were performed once.

### 3.2 Basic characterization

Dose reproducibility, dose linearity, dose rate dependency, PDD, and ROF measurements are shown in Figure 4. Maximum deviations in the results were compared to local department tolerances derived from the NCS 18 (2018) and AAPM task group 142 (Table 2).^[^ ^34^, ^35^ ^]^

**TABLE 2:** Summary of performance characterization measurements.

Test	Max difference	$u_{acr}$	Tolerance	Outcome
Short term reproducibility	−1.0%	±0.1%	1.5%	Passed
Medium term reproducibility	−0.9%	0%	1.5%	Passed
Dose linearity (1–2 MU)	−3.0%	±0.3%	5.0%	Passed
Dose linearity (5–2000 MU)	−1.4%	±0.1%	2.0%	Passed
Dose rate dependency	0.8%	±0.4%	2.0%	Passed
PDD (after Dmax)	−0.7%	±0.2%	1.0%	Passed
ROF (2.0–31.8 cm EqFS)	−3.2%	±2.0%	2.0%	Failed^*^
Axial dependence	2.5%	±0.1%	‐	‐
Orientation with magnetic field	10.8%	±2.1%	‐	‐

*Note*: The maximum deviation, influence of calibration $u_{acr}$, tolerance and outcome are shown.

^*^ for the mean $k_{acr}$, the relative output factor measurement exceeded the tolerance, but for some $k_{acr}$ it was within tolerance. See discussion.

The short term dose reproducibility had a standard deviation of σ = 0.5%, a maximum deviation of −1.0% and a calibration‐method dependency $u_{acr}$ of ±0.1%. The medium term dose reproducibility had a maximum readout deviation with respect to the mean dose of −0.9% ($u_{acr}$ = 0%) and an overall standard deviation of σ = 0.5%.

Dose linearity was measured for both the detector and Farmer type ionization chamber (Figure 4a). Over the whole range of MU, the mean dose difference of the scintillator with respect to the Farmer chamber was −0.7% ($u_{acr}$ = ±0.1%) with σ = 1.2%. All measurements were within tolerance (see Table 2), however a larger variation was observed relative to the Farmer chamber. The largest difference was found for the 1 MU measurement, which was ‐3.0% ($u_{acr}$ = ±0.3%).

The dose rate variation (Figure 4b) resulted in a mean difference in readout of 0.3 ± 0.3% (1 SD) with respect to the nominal dose rate of 420 MU/min. All measurements were within tolerance and the maximum deviation was 0.8% ($u_{acr}$ = ±0.4%) for the 252 MU/min measurement.

The PDD (Figure 4c) measured with the scintillation detector was in good agreement with the microDiamond. The mean difference with the microDiamond beyond $D_{max}$ was −0.1% ($u_{acr}$ = ±0.1%) with σ = 0.4%. The difference observed near the surface (−3 mm to +5 mm) was expected due to the difference between the two detector holders.^[^ ^36^ ^]^

ROFs were measured (see Figure 4d) for both the scintillator and the microDiamond detector. For small field sizes (2.0 × 2.0 cm^2^ to 7.0 × 7.0 cm^2^), the mean difference of the PSD with respect to the TRS‐483 corrected microDiamond reading was 1.4% ($u_{acr}$ = ±0.7%). For larger fields (starting from 15.0 × 15.0 cm^2^) this difference is up to −3.2% ($u_{acr}$ = ±2.0%) for field size 57.4 × 22.0 cm^2^. For the larger fields the Cherenkov contribution in *R*_1_ increases resulting in a decreased ratio of $R_{1,S}$ and $R_{1,C}$. This means that small errors in $k_{acr}$ leads to larger deviations. This was assessed by investigating the ROFs based on the $k_{acr}$ resulting from the square field method which shares a similar setup ($k_{acr}=1.09$, Table 1). In that case the differences of the scintillator compared to the microDiamond became smaller and the largest error at a field size of 57.4 × 22.0 cm^2^ was −1.9%.

### 3.3 Response of detector with respect to the magnetic field

The maximum effect of the axial rotation (defined in Figure 3a) on the response of the detector (see Figure 5a) was 2.5% ($u_{acr}$ = ±0.1%) at the 180° angle relative to 0°. This originates from a difference for the *R*_1_ and *R*_2_ readings (Figure 5b). Since the dose is the same, either the scintillation fiber is sensitive to the orientation of radiation or the assumption that change in Cherenkov contribution is the same in both channels is not valid for all conditions.

For the radial rotation (Figure 3b) a change in detector response was observed. The largest change in scintillator response was observed in the 67.5°–135.0° region with the maximum difference 10.8% ($u_{acr}$ = ±2.1%). For this region the variation in $k_{acr}$ also has the highest influence on $R_{1,S}$. As can be observed from Figure 6b, the Cherenkov signal is clearly higher for the angles 67.5°–135.0°. This demonstrates the asymmetric distribution of Cherenkov radiation in the magnetic field. Furthermore, the relative increase of *R*_1_ is different than *R*_2_, with a difference of 20.7% at 112.5°. This violates the assumption of equality, which is needed for proper working of the subtraction method for this detector.

### 3.4 The effect of MRI RF pulses and dynamic measurements

Time‐resolved measurements were performed for static (Figure 7d) and dynamic (Figure 7e) setup with and without MR image acquisitions. The four consecutive static measurements under different angles showed a mean dose difference of 0.4% ($u_{acr}$ = ±0.1%) with σ = 0.5% between the measurement with and without MRI acquisition. Measurements performed at 225° and 135° show lower *R*_1_ and *R*_2_ readings and dose compared to the readings at 315° and 45° (see Figure 7d) due to the couch transmission. It is noted that the cryostat transmission inhomogeneity is small.

Movement of the insert can be seen clearly in Figure 7b and 7c. The dynamic measurement showed a change in reading in both channels over time when the fiber was moved through the beam, resulting in a change in Cherenkov contribution (Figure 7e). This also suggests the same change in the Cherenkov contribution for both *R*_1_ and *R*_2_, as is required for proper working of the subtraction method. The resulting dose is however more or less constant since the motion of 2 cm is within the center of the field. For the dynamic measurement the mean dose difference is −0.6% ($u_{acr}$ = 0%) with σ = 0.3% between the measurement with and without MRI acquisition, which is similar to the static measurement.

## 4 DISCUSSION

The Blue Physics PSD relative dosimetry performance was assessed, with similar results to those of Ferrer et al.^[^ ^25^ ^]^ For the dose reproducibility, dose linearity, dose rate dependency and the PDD measurement, the influence of $u_{acr}$ was minimal.

Differences in ROFs between the scintillator and the microDiamond increase for field sizes ≥ 15.0 × 15.0 cm^2^, because the contribution of Cherenkov light changes relative to the scintillation reading for these large fields, and needs recalibration if the measurement setup is too different from the calibration setup. When using $k_{acr}$ of the ‘square field’ calibration, which is closer to the ROF measurement setup, the maximum difference is reduced. This indicates that in order to use the detector with these larger fields, the $k_{acr}$ would need to be recalibrated. The output correction factors for the measured microDiamond fields on which the analysis was performed (2.0 × 2.0 cm^2^ – 57.4 × 22.0 cm^2^) are all unity except the 2.0 × 2.0 cm^2^, with output correction factor 0.997.^[^ ^32^, ^33^ ^]^ Since this output correction factor is minor no large difference were found compared to uncorrected microDiamond readings.

Various calibration methods were investigated to determine $k_{acr}$ on the MR‐linac. More variation in the $k_{acr}$ calibration factor was observed for the MR‐linac than on the conventional linac. Ferrer et al. showed similar results for the square field calibration method ($k_{acr}$ = 1.09 and $k_{acr}$ = 1.08). The optimal calibration method for the Blue Physics detector should be further investigated, but the calibration conditions should be chosen such that they resemble the measurement setup, as can be observed in the ROF measurement. Otherwise, the assumption that the $k_{acr}$ is constant becomes invalid.

An axial rotation was observed for the detector. The measurement results (see Figure 5) agree well with the result obtained by Ferrer et al.^[^ ^25^ ^]^ It shows the limitation of the subtraction method which assumes that both channels have the same (constant ratio of) Cherenkov contribution such that any difference in reading can be attributed to a change in local dose. In this case the dose is the same, yet the $R_{1,S}$ reading varies, so either the scintillation fiber is orientation dependent or the ratio of Cherenkov contribution varies. The reason of this axial dependency is unknown. A potential explanation for the variation in Cherenkov contribution is a slight variation in fiber orientation between the two channels, since the Cherenkov contribution has a sharp demarcated orientational variation. When rotating the detector, the orientation of the fibers may vary relative to each other which may lead to a change in the ratio of Cherenkov contribution.

The scintillator response is dependent on its orientation with respect to the magnetic field and the photon beam, as seen in other detectors.^[^ ^24^, ^37^ ^]^ The reason of this dependence for this particular detector differs from ionization chambers and is currently unknown. Our hypothesis is that magnetic field influence on the electron fluence, which becomes asymmetric, also has an impact on the Cherenkov distribution. The largest increase in contribution of Cherenkov is obtained for the 67.5°–135.0° rotations. This is the direction in which electrons are bent by the Lorentz force and more Cherenkov light is expected. Also, the change in Cherenkov in both channels is different. This violates the assumption of constant Cherenkov contribution from both fibers and therefore the detector has to be recalibrated when positioned or rotated differently in the magnetic field.

Other studies which looked into the magnetic field dependence of fiber optical dosimeters observed reading differences when an increasing magnetic field was applied.^[^ ^21^, ^38^ ^]^ A study by Therriault‐Proulx et al. concluded that the Cherenkov contribution changed when a magnetic field was applied resulting in a different reading of the Exradin W1 (Standard Imaging, Middleton, Wisconsin, USA) scintillator.^[^ ^21^ ^]^ This is consistent with our hypothesis that due to the change in electron trajectories in a magnetic field also the Cherenkov radiation acquired in the fiber changes. For the subtraction method, this change in Cherenkov contribution should change in both fibers, resulting in a similar $k_{acr}$. Our hypothesis is that the increase in Cherenkov signal in both fibers is non linear which causes this behavior.

Furthermore, a spectral filtering based PSD characterized by Uijtewaal et al.^[^ ^39^ ^]^ for use in the 1.5 T MR‐linac also investigated the detector response while rotating the detector through the magnetic field. Their results showed only minor differences (0.3%–0.8%) when rotating the detector and seems able to correct for the change in Cherenkov distribution.

Detector radial dependence with respect to the magnetic field has been previously observed in, for example, Farmer ionization chambers,^[^ ^24^ ^]^ and might limit the setup possibilities. However, while the electron fluence is affected by the Lorentz force for both ionization chambers and PSDs, the effect on the reading has a different cause. Nevertheless, potentially similar limitations could apply to the use of this PSD.

MRI acquisition (RF pulse) dependency was investigated for both static and dynamic measurements and showed no difference between measurements with and without MR image acquisition. Therefore, the scintillation detector can be used for time‐resolved dose measurements during MR image acquisitions. This opens the possibility to perform real‐time dose measurements during MR guided gated delivery when the detector orientation with respect to the magnetic field is kept constant.

This is consistent with other studies on PSDs for use in a MR‐linac. Klavsen et al.^[^ ^16^ ^]^ demonstrated, using a different scintillation detector in a 0.35 T MR‐linac, that a PSD is capable of time‐resolved dose measurements. In their study, gating was investigated and the scintillation detector proved to be a powerful dosimeter for such treatments. Uijtewaal et al.^[^ ^39^ ^]^ also concluded that the scintillation detector in their study showed excellent results during MR scanning and dynamic measurements.

## 5 CONCLUSION

The Blue Physics scintillation system is found to be a capable relative dosimeter for high temporal resolution dose measurements during MRI acquisitions, in specific standardized configurations of the detector. However, a magnetic field impact on the response of the scintillator was observed. The orientation of the scintillator in the measurement setup should be close to the calibration setup, otherwise the assumption that the ratio of Cherenkov contribution in both channels is constant is not valid. This implies that the scintillator has to be calibrated for the specific orientation relative to the magnetic field. Further investigation is needed for the optimal calibration method for the MR‐linac.

A key result of this study is that dynamic time‐resolved dosimetry can be performed with this PSD since it was not affected by motion within the magnetic field or by MR image acquisition, when keeping its orientation relative to the magnetic field constant. This shows to be a very useful property of this detector.

## CONFLICT OF INTEREST STATEMENT

The authors have no conflict of interest to disclose.
